# Space-Use Patterns of the Asiatic Wild Ass (*Equus hemionus*): Complementary Insights from Displacement, Recursion Movement and Habitat Selection Analyses

**DOI:** 10.1371/journal.pone.0143279

**Published:** 2015-12-02

**Authors:** Nina Giotto, Jean-François Gerard, Alon Ziv, Amos Bouskila, Shirli Bar-David

**Affiliations:** 1 Mitrani Department of Desert Ecology, Jacob Blaustein Institutes for Desert Research, Ben-Gurion University of the Negev, Midreshet Ben-Gurion, Israel; 2 Comportement et Ecologie de la Faune Sauvage, Institut National de la Recherche Agronomique, Castanet-Tolosan Cedex, France; 3 Department of Life Sciences, Ben-Gurion University of the Negev, Beer-Sheva, Israel; University of Sydney, AUSTRALIA

## Abstract

The way in which animals move and use the landscape is influenced by the spatial distribution of resources, and is of importance when considering species conservation. We aimed at exploring how landscape-related factors affect a large herbivore’s space-use patterns by using a combined approach, integrating movement (displacement and recursions) and habitat selection analyses. We studied the endangered Asiatic wild ass (*Equus hemionus*) in the Negev Desert, Israel, using GPS monitoring and direct observation. We found that the main landscape-related factors affecting the species’ space-use patterns, on a daily and seasonal basis, were vegetation cover, water sources and topography. Two main habitat types were selected: high-elevation sites during the day (specific microclimate: windy on warm summer days) and streambed surroundings during the night (coupled with high vegetation when the animals were active in summer). Distribution of recursion times (duration between visits) revealed a 24-hour periodicity, a pattern that could be widespread among large herbivores. Characterizing frequently revisited sites suggested that recursion movements were mainly driven by a few landscape features (water sources, vegetation patches, high-elevation points), but also by social factors, such as territoriality, which should be further explored. This study provided complementary insights into the space-use patterns of *E*. *hemionus*. Understanding of the species’ space-use patterns, at both large and fine spatial scale, is required for developing appropriate conservation protocols. Our approach could be further applied for studying the space-use patterns of other species in heterogeneous landscapes.

## Introduction

The way in which animals move and use the landscape is influenced by the spatial distribution of resources such as forage, water and shelter [1–7]. One way to identify the relevant factors is to investigate habitat selection by comparing habitat use with availability [8]. Another way is to analyse the animals’ movements within their home ranges, assuming individuals return more often to preferred sites [9,10]. Recursion is defined as the return by animals to previously visited sites [11–14]. The level of recursion of herbivores to a feeding site is assumed to influence vegetation renewal [15] and, according to optimal foraging theory, should be a function of the site’s quality, the duration of last use, and the time elapsed since last use [11,16,17]. However, recursion movements may also occur to resources that are potentially less dependent on the individuals’ behaviour, such as water sources [18] and favourable resting sites [19]. Recursions to particular sites might occur at similar times of the day [13] leading to 24-hour periodicity in recursion patterns. Characterizing recursion sites—in terms of habitat, landscape characteristics, and time interval among recursions—may thus help to identify the different factors that influence movement patterns [11,13], and may provide complementary insights to the habitat selection approach [20].

In the present paper, we explore how the spatial distribution of resources and habitat-related factors affect the space-use patterns of a large mammalian herbivore, by combining the two aforementioned approaches: habitat selection and movement analyses. We also develop simple methods to identify the most frequently visited sites (i.e. high recursion sites), and to detect whether animals tend to return to the same areas at the same time of the day.

We studied the Asiatic wild ass (*Equus hemionus*), an endangered equid inhabiting deserts and mountain steppes [21–26]. Few studies have investigated the species’ movements and habitat selection [26–31], and none of them at the home range level (3^rd^ order selection *sensu* Johnson [32]). A better understanding of the species’ space-use patterns, at both the large and fine spatial scale, is required for developing appropriate conservation protocols. Accordingly, in the present study carried out in the Negev Desert, Israel, we used GPS radio collars recording the locations of wild asses at one-hour intervals in the course of two seasons (summer and winter), and analysed how habitat selection, the distances travelled, and recursion patterns varied in the course of the 24-h cycle and between seasons.

In arid environments, vegetation is scarce and heterogeneously distributed [33]. During the driest season—summer in the Negev Desert—there is no or only negligible growth in most annual and perennial plants, and herbivores face nutritional bottlenecks [34]. In the same season, water is also a critical resource [35–38], especially for water-dependent species [39–41]. Finally, non-burrowing mammals are exposed to high temperatures during the hottest hours of summer, and in such conditions, they tend to reduce the time they spend active (decreasing feeding time and movements) and/or remain in cooler microhabitats [42–47].

The Asiatic wild ass being a mobile and water-dependent herbivore [28,40,48], we expected that the monitored individuals (1) had larger ranges and (2) travelled greater distances in summer than in winter to cover their nutritional and drinking needs. We further expected that in summer, because of the high temperatures during midday hours, (3) activity was minimized during the hottest hours, (4) distances travelled were shorter during the daylight hours than during the night, (5) high-elevation areas, which are relatively windy, and north-facing slopes, which receive less sun radiation, were selected during the midday hours, and (6) high vegetation cover was selected during the night. Because habitat selection might exhibit a daily pattern, we expected (7) to find a 24-h periodicity in recursion times (duration between visits). Finally, due to the seasonal variation in vegetation renewal, water constraints and climatic conditions, we expected (8) the recursion rate to differ between seasons and (9) recursions to occur on different types of sites, the main summertime recursion sites being favourable resting sites rather than favourable feeding sites.

## Material and Methods

### Study species

The Asiatic wild ass was once abundant in western Asia, including the Negev Desert, Israel [49]. As is the case for many other large mammalian herbivores (e.g. [50–54]), the species has declined throughout its range due to hunting and habitat loss [40,55], and the Asiatic wild ass is now classified as endangered by the IUCN Red List [25]. The subspecies endemic to the Middle East (*E*. *h*. *hemippus*) became extinct in the early 20th century [56]. In 1968, a captive breeding program was initiated with 11 individuals from the subspecies *E*. *h*. *onager* and *E*. *h*. *kulan*, which are typically found in Iran and Turkmenistan, respectively. Between 1982 and 1993, the Israel Nature and Parks Authority (INPA) reintroduced some of the captive animals into Makhtesh Ramon and the Paran Wadi within the Negev Desert. During the 90s, the population expanded its range to the Negev Highlands and the Arava Valley [21]. It is now estimated at *ca*. 250 individuals [31,57].

### Study area

**Fig 1:**
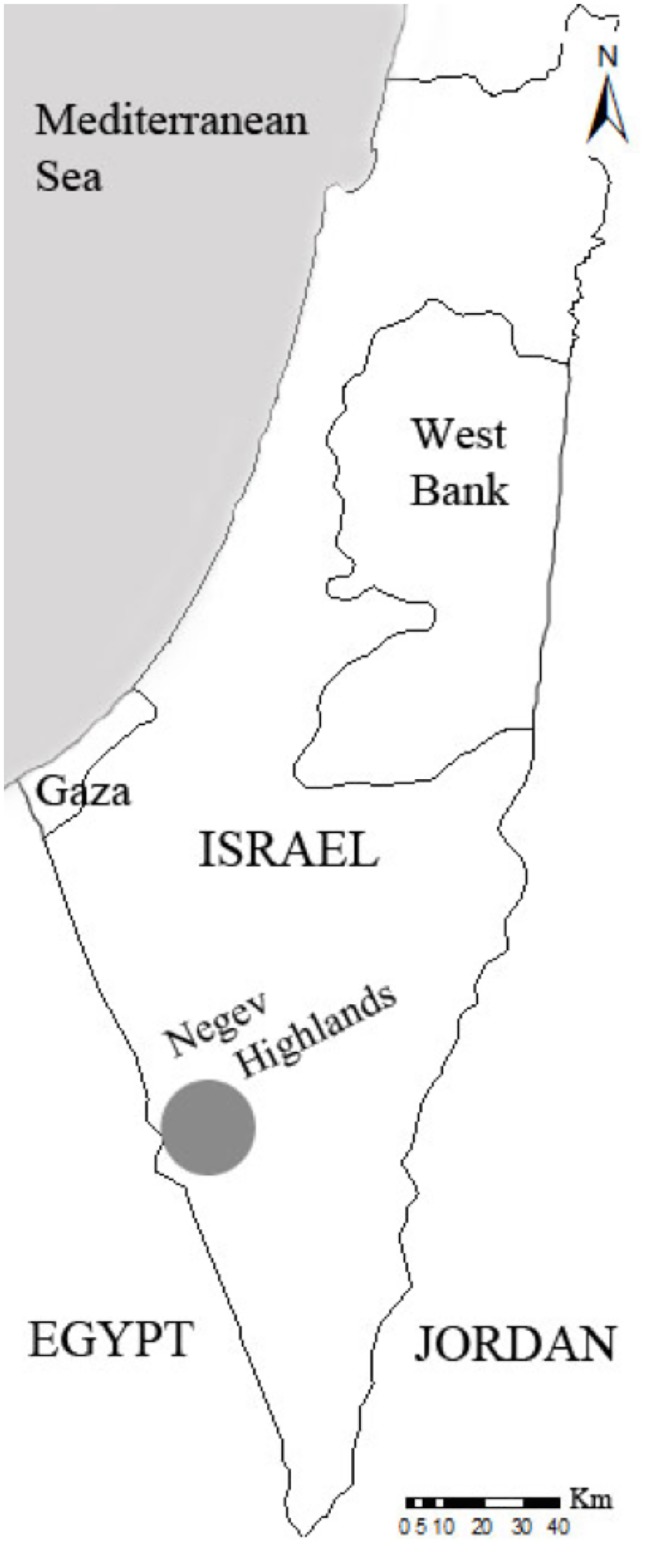
Study area location (grey spot) within the Negev Highlands, southern Israel.

The study was carried out in the Negev Highlands (Fig 1), in a 300-km^2^ area of rocky hills (30°32’N; 34°38’E) delimited by the Egyptian border to the west, and the Makhtesh Ramon erosion cirque to the southeast. Main bedrocks are limestone, chalk, marl and dolomite, often covered by loess. Elevation ranges from 470 to 1037 m above sea level. The climate is arid, with hot and very dry summers. Maximum and minimum temperatures average 30.7 and 18.4°C in July and August (hottest months), and 12.7 and 5.9°C in January (coldest month; [58]). However, convective winds [59], inducing a wind chill effect in high-elevation areas, are common all year long because of the daytime heating of the Makhtesh Ramon. Annual precipitation ranges from 30 to 150 mm, more than 90% of which occurs between October and April [58,60]. When concentrated rain events occur, water become temporarily available in ancient Nabataean cisterns, and natural holes located in the thalwegs (streambeds) fill with water that may remain until July [40]. Because no substantial permanent water source existed in the area, an artificial source (hereafter referred to as ‘the artificial water point’) was set up by the INPA during the 1990s to provide year-round access to water. This water point is the main permanent water source in the Negev Highlands and was used by a large number of wild asses during the study. However, our data led us to discover that two additional water sources were also used, a pipe that leaked and created a small puddle (hereafter referred to as ‘the leaking pipe’), and the water point of an isolated farm (hereafter ‘the alpaca farm water point’) located outside the area typically used by the wild asses of the Negev Highlands’ population. Trees (*Pistacia atlantica*) are very sparse in the landscape; vegetation is of Saharo-Arabian type and mostly present along thalwegs in summer [33]. Rather rare groups of dromedaries (*Camelus dromedarius*) roam the hills. The only wild ruminants are the Dorcas gazelle (*Gazella dorcas*) and the Nubian ibex (*Capra nubiana*) in the cliffs. The largest carnivores in the area are the striped hyena (*Hyaena hyaena*), the Arabian wolf (*Canis lupus arabs*), and possibly the critically endangered Arabian leopard (*Panthera pardus nimr*) [61].

### Data collection

Location data were collected using the global positioning system (GPS) monitoring technique. Five wild asses (four males: individuals 594, 595, 596, and 597; one female: individual 598) were captured near the artificial water point between July 2012 and early August 2013. The individuals were shot from a shelter located above the water point, using a dart gun (J-M 25 Special by Dan-inject). They were immobilised by a mixture of Etorphine Hydrochloride (Captivon^™^ 98 by Wildlife Pharmaceuticals) and Butorphanol (Butomidor^®^ 10mg/ml). The five animals were equipped with GPS collars (African Wildlife Tracking company) that were scheduled to attempt a location every hour. After the procedure of collaring, Naltrexone (Trexonil^™^ 20 50 mg/ml by Wildlife Pharmaceuticals) was injected IV to antagonise the Etorphine. A permit to capture and collar the wild asses was given by the Israel Nature and Park Authority, so that all necessary permits were obtained for the described study, which complied with all relevant regulations.

In addition to GPS location data, we collected data on the species activity rhythm. Visual observations were made in summer, using the scan sampling method [62]: for any group encountered while driving around in the study area, we chose an individual at random (among the collared members, if any), and recorded its activity every five minutes. The individual was considered as resting when lying or standing motionless; otherwise, it was considered as active. A total of 274 hours of observation was performed.

### Data analysis

Two 50-day periods that provided almost complete sequences of the GPS locations for the five equipped animals were used for the present study: 12 Aug.–30 Sep. 2013 for summer, and 12 Dec. 2013–30 Jan. 2014 for winter.

#### Fifty-day home ranges

The monitored individuals’ 95% and 50% fifty-day home ranges (here and after defined as home range—HR) were estimated for each season, using the Movement-based Kernel Density Estimator (MKDE; [63,64]). In contrast to the classical kernel estimators, MKDE takes into account the correlation between successive locations. Estimation was performed with the following parameter values: *T*_*max*_ = 2.5 hour, τ = 0.1 hour, *L*_*min*_ = 50 m, *h*_*min*_ = 100 m, *b* = 0 (see [64,65] for details).

#### Displacement

We computed the distances between successive locations for each monitored animal within a given season. For each individual and season, we then calculated the mean of the distances obtained for each hour of day. The effects of season and hour of day on this mean distance were analysed using linear mixed-effect models, in which the season and hour were set as fixed-effect categorical variables, and the individual as a random-effect factor.

#### Visits to the ‘permanent’ water points

In order to assess the number of visits to a water point on the basis of GPS data, we began by estimating the mean distance travelled per hour by individuals visiting the water points. So, we computed the distance between successive GPS-locations for each series composed of at least three locations passing within 50 m of any of the three known water points (all the locations of a series but the first and the last were at a distance of less than 50 m). Mean distance travelled per hour (± SE) appeared to differ between water points (artificial water point: 879 ± 48 m; leaking pipe: 408 ± 36 m; alpaca farm water point: 1,633 ± 257 m; F_2,221_ = 24.522, P < 0.0001). On the basis of these results, any location or sequence of consecutive locations situated inside a circle of a 440-m radius (880-m diameter) centred at the artificial water point, a circle of a 210-m radius (420-m diameter) centred at the leaking pipe, or a circle of a 820-m radius (1,640-m diameter) centred at the alpaca farm water point was considered as a visit to the corresponding water point. In the case of a sequence of locations inside the circle, the time of the visit was the time of the closest location to the water point.

#### Habitat selection

Habitat was characterized by five landscape-related variables that could be obtained or computed from geographic information system (GIS) layers: (1) thalweg vs non-thalweg area, (2) normalized difference vegetation index (NDVI), (3) elevation, (4) slope, and (5) northern exposure. As the hydrological network was encoded in the form of lines in ArcGIS (http://mapi.gov.il/en/Pages/default.aspx), thalweg areas were identified, applying a 25-m buffer to this network; the corresponding variable took value 1 inside the buffer and 0 outside. NDVI scores were derived from Landsat 8 data (30 x 30 m resolution) taken on 6 Sep. 2013 for summer and on 12 Jan. 2014 for winter. The NDVI does not provide any information on vegetation type, but the wild ass is considered as very flexible with respect to plant community [26,28,66]. Elevation above sea level and slope (in degrees) were derived from a 25 x 25 m resolution digital elevation model (DEM) of the Negev Desert. Aspect (i.e., slope azimuth, measured clockwise in degrees from the northern baseline) was extracted from the same model and converted into the variable ‘northern exposure’:

$$E_{N}=\text{sin}\theta \text{cos}\alpha ,$$

where *θ* is the slope, and *α*, the slope azimuth. *E*_*N*_ is the orthogonal projection of the unit vector that is perpendicular to the slope on the south-north axis. Its value ranges from –1 for a south-facing vertical slope to +1 for a north-facing vertical slope. Preliminary analyses showed that the habitat variables retained were only weakly correlated over the study area (the absolute value of Pearson’s correlation between two of them never exceeding 0.374 over a set of 1,135 points drawn at random).

The five habitat variables were computed for each location falling into the individual’s 95% fifty-day home range, as well as for each node of a 30-m square grid covering the same home range. Mean and SD of the values obtained for the nodes were calculated for each habitat variable and home range. The same was done with the values obtained for the locations, but for each hour of day. For each habitat variable, and for each season, hour of day and individual, we then computed the selection coefficient

$$SC=\frac{\text{location mean}-\text{node mean}}{\text{node SD}}.$$

The effects of season and hour of day on the value of *SC* were analysed using linear mixed-effect models similar to those used for the displacements. The same models were further used for testing whether *SC* differed from 0 (Student’s t-test on the estimated values of *SC*).

#### Residence and recursion

In relation to the median and mean distances travelled by an individual per hour (183 and 372 m, respectively), we computed for each location (1) the number of consecutive hours the individual was located within a 200-m radius (‘residence time’), (2) the number of hours it was located beyond this radius before returning (‘recursion time’), and (3) the number of times the individual entered the so-defined circle during the 50 days (‘number of visits’). As many locations were not revisited during the 50-day sampling periods, no mean was calculated for recursion time. Nevertheless, the proportion of revisited locations was sufficient for estimating median recursion times. This was done, using the Kaplan–Meier method [67] to compute the ‘survival curve’ of recursion times, and then recording the time for which survival was equal to 50%.

In order to examine whether the animals tended to return to the same sites at the same hours of day, we assumed that the number of recursions recorded for any duration *t* followed a Poisson law, the parameter λ of which was a function of *t*. Under this assumption, we fitted the data with a series of generalized linear models (family: Poisson), either including or not including oscillations of a 24-h period (Table 1), and compared them using Akaike information criterion (AIC; [68]).

**Table 1:** AIC value of the generalized linear models (family: Poisson) fitted to the summer and winter distributions of recursion times. Models M1 to M3 do not oscillate with time. Models M4-k and M5-k include a cosine (or log-cosine) function of 24-h period, and a parameter (k) specifying the time at which the first maximum occurs; they reduce to model M3 when α_3_ = 0; their respective AIC values were calculated for all the integer values of k between 0 and 23. The smallest AIC value was obtained with model M5-k for summer and model M4-k for winter, in both cases for k = 3, implying that the envisaged models that best fit the data exhibited maximums every 24 h minus 3 h.

Models		df	AIC summer	AIC winter
M1	log_*e*_(λ) = α_0_ + α_1_ *t*	2	4283.8	4141.4
M2	log_*e*_(λ) = α_0_ + α_1_log_*e*_(*t*)	2	3707.5	3784.1
M3	log_*e*_(λ) = α_0_ + α_1_ *t* + α_2_log_*e*_(*t*)	3	3276.4	3510.5
M4-k	log_*e*_(λ) = α_0_ + α_1_ *t* + α_2_log_*e*_(*t*) + α_3_cos[2π(*t*+k)/24]	4	2440.3–3276.4	3226.8*– 3511.6
M5-k	log_*e*_(λ) = α_0_ + α_1_ *t* + α_2_log_*e*_(*t*) + α_3_log_e_{cos[2π(*t*+k)/24]+2}	4	2416.8*– 3276.5	3231.9–3512.0

λ: Poisson law parameter;

*t*: time in hours; k: integer ranging from 0 to 23;

α_0_, α_1_, α_2_, α_3_: fitted parameters;

*: best AIC value for the season.

We analysed the effects of the season and home range size on the mean number of visits per location for each individual and season. As mean location density is inversely proportional to home range size, we expected the same relationship between the mean number of visits per location and home range size. Such a relationship becomes linear with a slope of -1 when the involved variables are log-transformed. Accordingly, the analysis was performed fitting a series of linear mixed-effect models (Table 2), in which the mean number of visits per location and home range size were log-transformed, whereas the season was set as a fixed-effect categorical variable, and the individual as a random-effect factor. The models were fitted using maximum likelihood and compared using AIC corrected for small samples (AICc; [68]).

**Table 2:** AICc value of the linear mixed-effect models considered for analysing the effects of the season and home range size on the mean number of visits per location.

Models (fixed part)		df	AICc 95% HR	AICc 50% HR
R1	log_*e*_(*ñ*) = α_0_^season^ + α_1_^season^log_*e*_(*A*)	6	35.5	27.2
R2	log_*e*_(*ñ*) = α_0_ + α_1_^season^log_*e*_(*A*)	5	21.6	19.2
R3	log_*e*_(*ñ*) = α_0_^season^ + α_1_log_*e*_(*A*)	5	25.4	16.4
R4	log_*e*_(*ñ*) = α_0_^season^–log_*e*_(*A*)	4	16.4*	9.1*
R5	log_*e*_(*ñ*) = α_0_^season^	4	34.6	34.6
R6	log_*e*_(*ñ*) = α_0_ + α_1_log_e_(*A*)	4	32.2	18.8
R7	log_*e*_(*ñ*) = α_0_ –log_e_(*A*)	3	27.3	13.7
R8	log_e_(*ñ*) = α_0_	3	36.5	36.5

*ñ*: mean number of visits per location;

*A*: home range size;

α_0_^season^, α_1_^season^: fitted parameters depending on the season;

α_0_, α_1_: fitted parameters independent of the season;

*: best AIC value.

#### Characterizing main recursion sites

For each individual and season, we mapped the 20% of locations that had the highest number of visits. Every cluster of at least 15 locations whose circles of 200-m radius overlapped was considered as a ‘main site of recursion’. Each site was characterized by the means and SDs of the five habitat variables computed over its locations, as well as the time of the day at which the locations were recorded. We might *a priori* have chosen any threshold between 10% and 50% to define the main recursion sites. With our dataset, however, mapping 20% of the locations appeared as the best compromise: using a higher percentage resulted in the inclusion of sites with very low recursion levels in winter, whereas using a lower percentage generated sites composed of too few locations to be characterized.

#### Software and descriptive statistics

Ecological characteristics of the GPS locations and home ranges were obtained using ArcGIS10 (ESRI, Environmental Systems Research Institute, Inc., Redlands, California, USA). Statistical calculations were performed using the R software (R Core Team 2014) and, more specifically, the ‘adehabitatHR’ package [65] for the MKDE method, the ‘nlme’ package [69] for the linear mixed-effect models, and the ‘survival’ package [70] for the Kaplan–Meier method. In the Results section, unless otherwise specified, means are given ± SE. In the case of repeated measures per individual, this refers to the mean ± SE of the individual means.

## Results

### Fifty-day home ranges

Contrary to what was expected, 50-day home ranges were not larger in summer than in winter (one-sided paired-sample t-test; 95% HR: t_4_ = -0.891, P = 0.79; 50% HR: t_4_ = -2.030, P = 0.94), but actually tended to be smaller in summer (summer vs. winter; 95% HR: 23.56 ± 5.29 km^2^ vs 31.27 ± 7.06 km^2^; 50% HR: 3.01 ± 0.90 km^2^ vs 7.28 ± 2.01 km^2^).

In summer, the five 50% home ranges included a ‘permanent’ water point, either the artificial water point (four animals) or the leaking pipe (individual 594). In winter, only individual 594 had a ‘permanent’ water point (the leaking pipe) in his 50% home range. Overall, the arithmetic centres of the individuals’ locations were closer to the nearest water point in summer than in winter (1.4 ± 0.3 km vs 5.0 ± 0.6 km; paired-sample t-test: t_4_ = -4.614, P = 0.01), the alpaca farm water point being always the farthest (summer: 15.6 ± 1.0 km; winter: 17.6 ± 2.3 km).

### Visits to the ‘permanent’ water points

**Fig 2:**
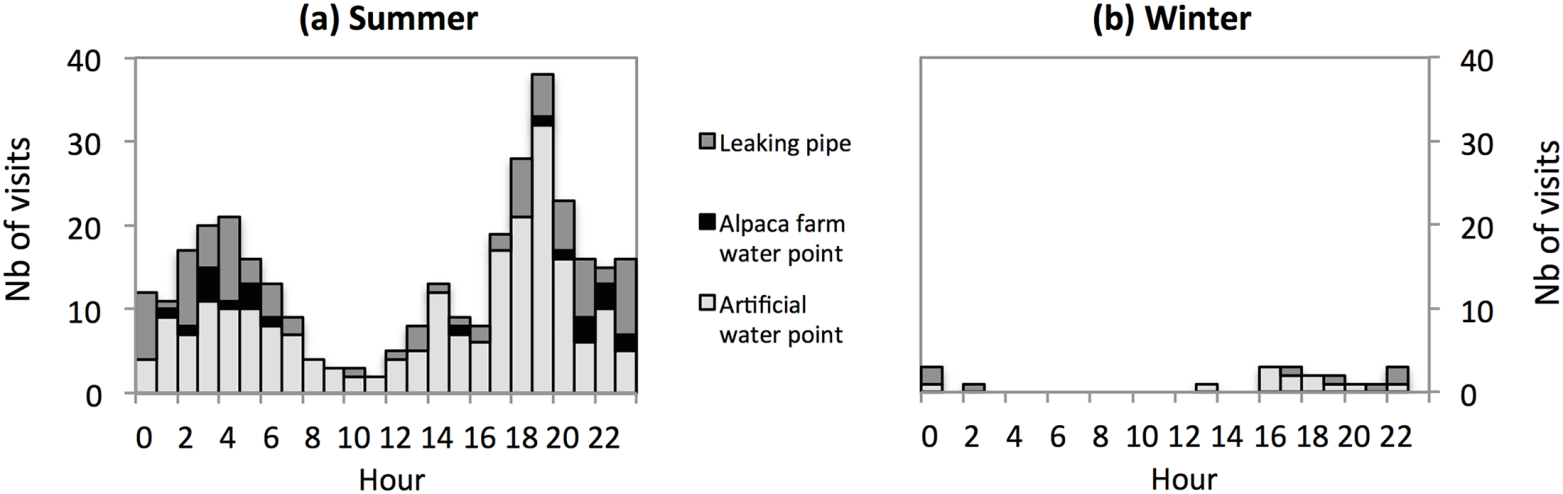
Number of visits to the ‘permanent’ water points as a function of the hour of day.

On average, an individual visited a water point 1.33 ± 0.17 times per day in summer and 0.08 ± 0.05 times per day in winter (paired-sample t-test: t_4_ = 8.430, P = 0.001). The artificial water point was visited at various times of the 24-hour cycle with a major peak at dusk in summer, and mostly during night-time in winter (Fig 2). The leaking pipe was nearly exclusively visited by individual 594 and at night in both seasons. The alpaca farm water point was only used in summer, mostly during a 10-day period while the artificial water point was dry; it was visited at night, and never by individual 594.

### Displacement and activity

#### Distance

**Fig 3:**
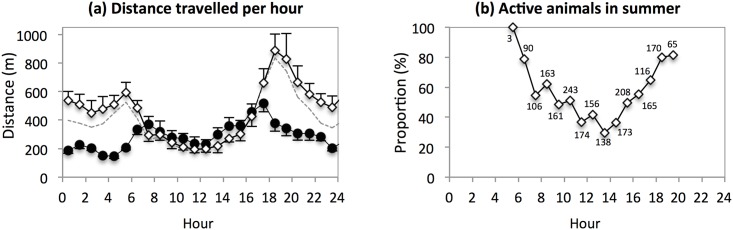
Displacement and activity: (a) Mean distance travelled per hour (and SE) as a function of the hour of day, in summer (white diamonds) and winter (black circles); dotted line: mean distance travelled per hour in summer when excluding the days with trips to the alpaca farm water point. (b) Proportion of scans for which the observed individuals were active as a function of the hour of day in summer (scan number is given for each proportion).

Distance travelled per hour varied throughout the day in summer (F_23,92_ = 7.307, P < 0.0001) and winter (F_23,92_ = 9.533, P < 0.0001), with peaks at dawn and dusk in both seasons (Fig 3a). However, the hourly patterns differed between seasons (F_23,188_ = 6.362, P < 0.0001), and overall, the distance travelled per hour was greater in summer than in winter (452 ± 28 m vs 291 ± 32 m; F_1,211_ = 61.872, P < 0.0001). Indeed, whereas the mean distance covered per hour differed slightly between seasons during the daylight hours (8:00–16:00; summer: 243 ± 26 m; winter: 294 ± 40 m), it was greater in summer than in winter at night (20:00–4:00; 529 ± 68 m vs 234 ± 24 m) and twilight (dawn peak: 591 ± 73 m vs 369 ± 58 m; dusk peak: 887 ± 117 m vs 518 ± 59 m). The day-night contrast recorded in summer persisted when excluding the 24-h cycles with trips to the distant alpaca farm water point (Fig 3a; 8:00–16:00: 236 ± 26 m; 20:00–4:00: 407 ± 39 m), even though these trips, mainly performed at night, provided the greatest distances travelled per hour (up to 5,284 m).

#### Activity rhythm

The individuals observed in summer were generally active at dawn and dusk and mostly inactive during midday. Furthermore, the proportion of scans for which they were observed in activity between 5:00 and 20:00 (Fig 3b) was positively correlated to the mean distance travelled per hour by the GPS-equipped animals (Fig 3a; Spearman’s correlation: r_s_ = 0.85, n = 15, P < 0.0001).

### Habitat selection

#### NDVI

**Fig 4:**
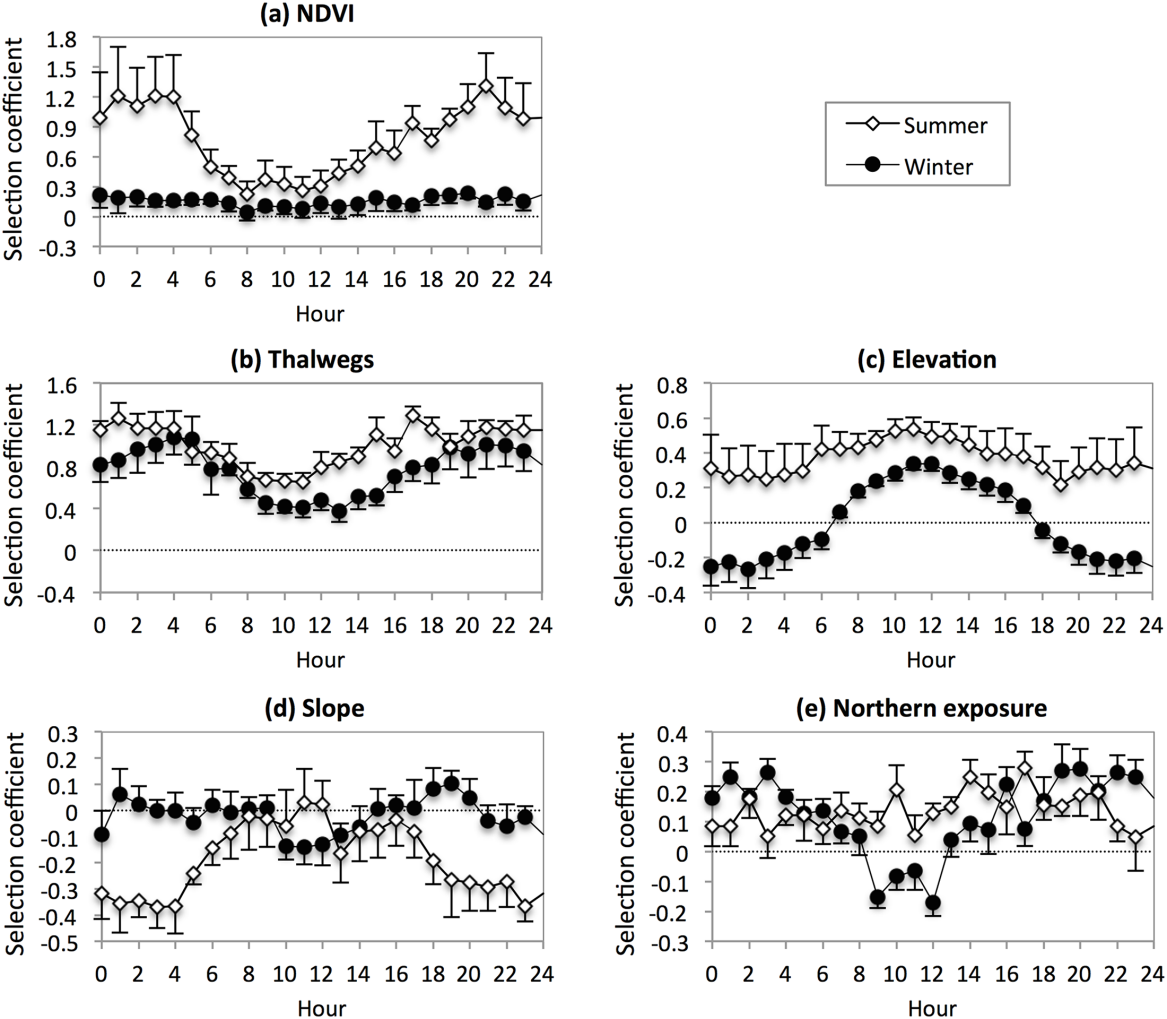
Mean selection coefficient (and SE) as a function of the season and hour of day, for each habitat variable.

On average, the individuals’ 95% home ranges had lower NDVI scores in summer than in winter (0.109 ± 0.001 *vs* 0.129 ± 0.002). Hourly patterns (F_23,188_ = 1.730, P = 0.025) and the overall intensity of selection (F_1,211_ = 136.629, P < 0.0001) differed between seasons (Fig 4a). In summer, individuals selected areas with high NDVI scores during the night (hour effect: F_23,92_ = 2.570, P < 0.001; t-test on the estimated values of *SC*: P < 0.05 from 15:00 to 5:00). In winter, the *SC* value was independent of the hour of day (F_23,92_ = 0.600, P = 0.92) and almost equal to 0 (t_4_ = 2.367, P = 0.08).

#### Thalwegs

On average, 50.9 ± 2.1% of the individuals’ locations were in thalweg areas, whereas these areas only covered 18.6 ± 0.6% of the seasonal 95% home ranges. Hourly patterns of selection intensity did not differ between seasons (Fig 4b; F_23,188_ = 0.704, P = 0.84), but the selection of thalweg areas was stronger in summer than in winter (F_1,211_ = 38.062, P < 0.0001), as well as stronger during the night than during the day in both seasons (hour effect: F_23,211_ = 4.668, P < 0.0001).

#### Elevation

Mean elevation of the individuals’ 95% home ranges was 924 ± 14 m in summer, and 883 ± 9 m in winter. Hourly patterns did not differ significantly between seasons (Fig 4c; F_23,188_ = 1.146, P = 0.30). Individuals tended to select higher elevations in summer than in winter (F_1,211_ = 179.932, P < 0.0001), as well as during the day than during the night in both seasons (hour effect: F_23,211_ = 5.282, P < 0.0001; t-test on the estimated values of *SC*: P < 0.05 at all hours in summer, from 9:00–13:00 and at 2:00 in winter).

#### Slope

Mean slope of the individuals’ 95% home ranges was 7.5 ± 0.4° in summer, and 10.7 ± 0.3° in winter. Hourly patterns (F_23,188_ = 1.997, P = 0.006) and the overall intensity of selection (F_1,211_ = 39.087, P < 0.0001) differed significantly between seasons (Fig 4d). In summer, individuals tended to select areas with low slopes during the night (hour effect: F_23,92_ = 1.997, P = 0.011; t-test on the estimated values of *SC*: P < 0.05 from 19:00 to 5:00). In winter, no consistent selection was detected despite a tendency of the animals to use slopes lower than expected by chance during the midday hours (hour effect: F_23,92_ = 1.083, P = 0.38; t-test on the estimated values of *SC*: P < 0.05 at 10:00–11:00).

#### Northern exposure

Overall, the individuals’ 95% home ranges were slightly north-exposed (mean value of E_N_; summer: 0.011 ± 0.003; winter: 0.013 ± 0.002). Hourly patterns differed significantly between seasons (Fig 4e; F_23,188_ = 3.432, P < 0.0001) but not the overall intensity of selection (F_1,211_ = 0.688, P = 0.41). In summer, north-exposed slopes tended to be selected throughout the 24-h cycle (hour effect: F_23,92_ = 0.968, P = 0.51). In winter, the same exposure was selected during the night, but not during the daylight hours, the monitored individuals tending to use areas less north-exposed than expected by chance from 9:00 to 12:00 (hour effect: F_23,92_ = 6.375, P < 0.0001; t-test on the estimated values of *SC*: P < 0.05 from 18:00 to 6:00, and at 9:00 and 12:00).

### Residence and recursion

#### Residence and recursion times

**Fig 5:**
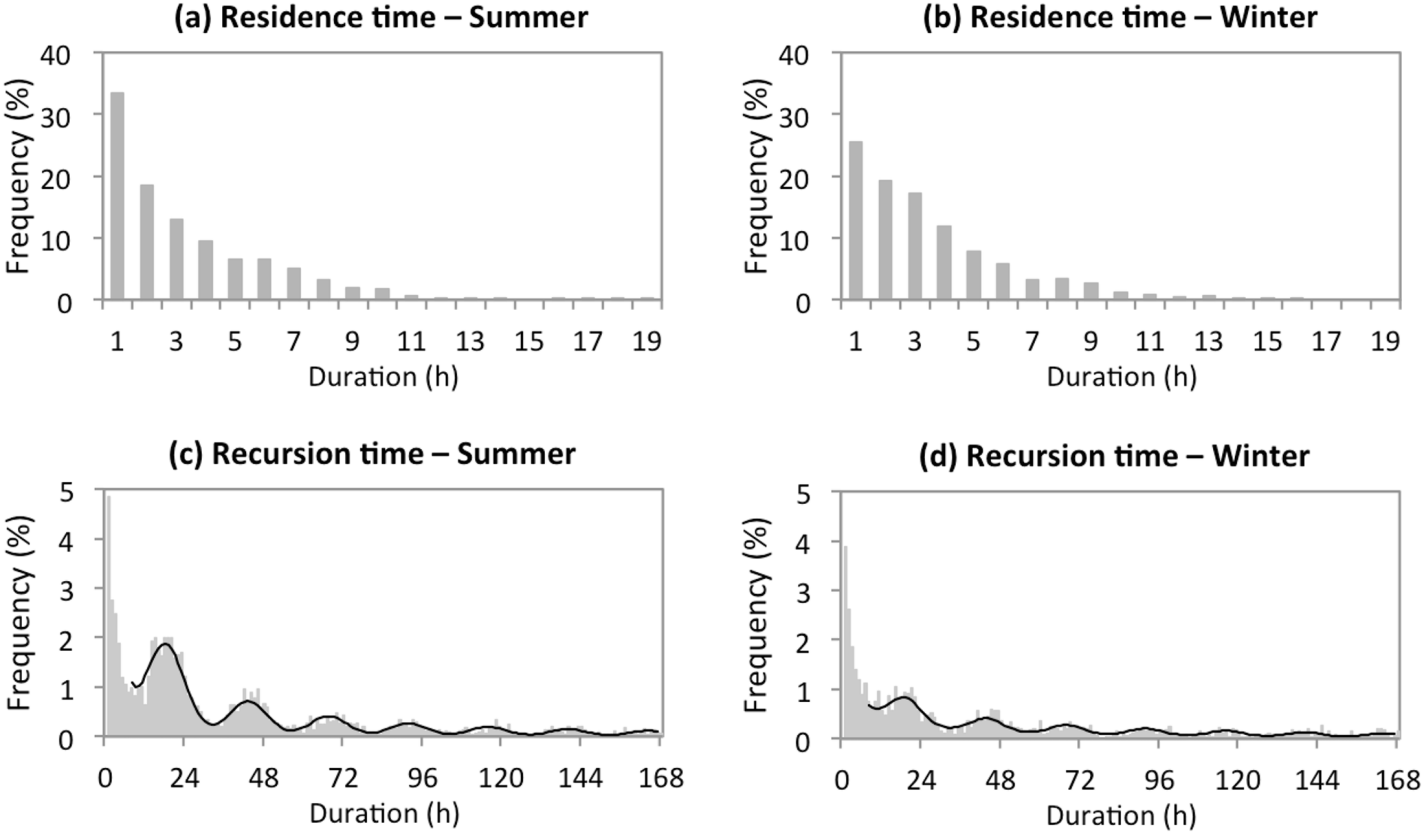
Overall distribution of residence and recursion times in summer and winter. Recursion times are shown for seven days (168 h). In (c) and (d), black curves correspond to the models of Table 1 that best fit the data (models M5-k and M4-k, respectively, with k = 3 in both cases).

Residence time did not differ significantly between seasons (Fig 5a and 5b; summer: 3.29 ± 0.29 h; winter: 3.50 ± 0.28 h; paired-sample t-test on the individual means: t_4_ = -2.382, P = 0.076). In contrast, recursion time was significantly shorter in summer than in winter (mean of the individual medians; summer: 2.49 ± 0.86 days; winter: 13.28 ± 4.27 days; paired-sample t-test on the individual medians: t_4_ = -2.804, P = 0.049). In both seasons, the distribution of recursion times exhibited peaks for durations that were a multiple of 24 h minus 3 h (Fig 5c and 5d; Table 1). Mean residence time being approximately 3 h, this implies that the individuals tended to return to certain sites at the same times of the day.

#### Recursion rate

**Fig 6:**
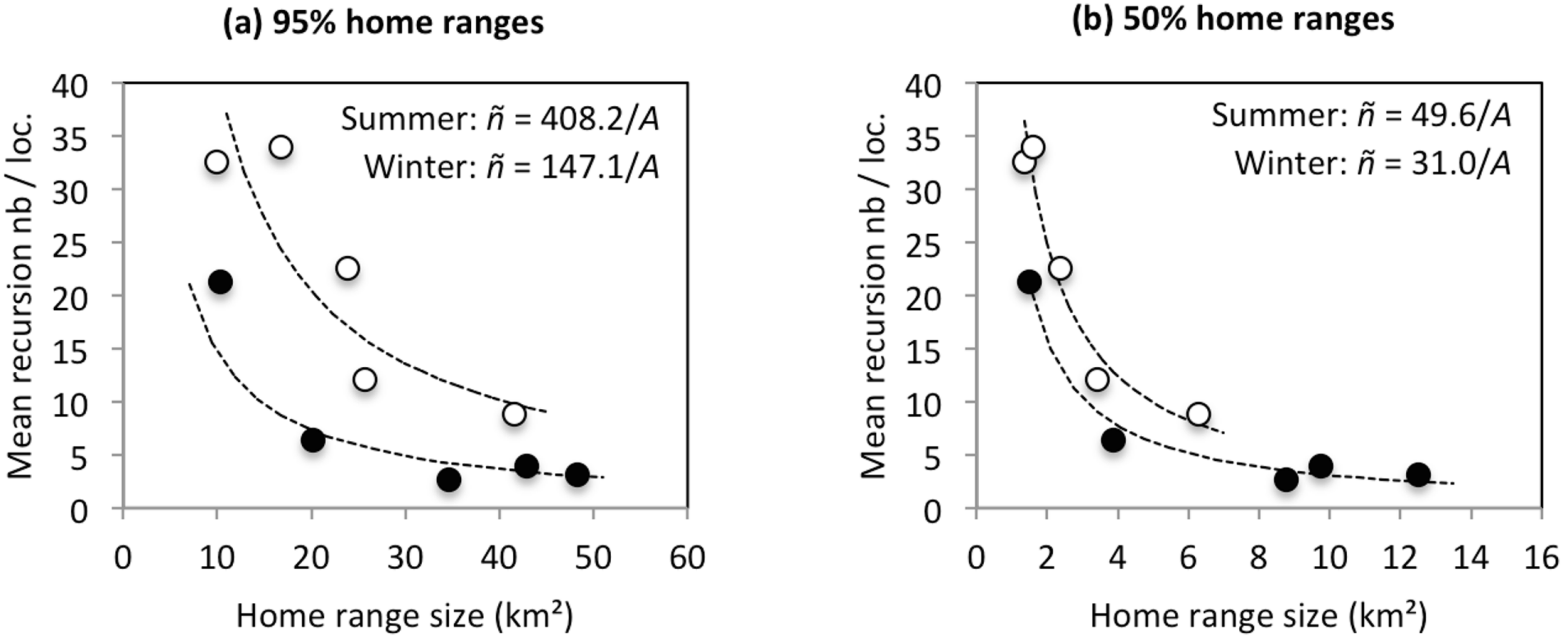
Mean number of visits per location as a function of the size of the 95% and 50% home ranges. White circles: summer HR; black circles: winter HR. In each graph, the curves correspond to the model of Table 2 that best fits the data (model R4 in both cases).

In accordance with the result obtained for median recursion time, the mean number of visits per location was higher in summer than in winter (22.00 ± 5.13 *vs* 7.51 ± 3.51; paired-sample t-test on the individual means: t_4_ = 3.339, P = 0.029). As expected, it tended to be inversely proportional to home range size; however, it remained greater in summer than in winter when home range size was taken into account (Fig 6 and Table 2).

#### Main recursion sites

**Fig 7:**
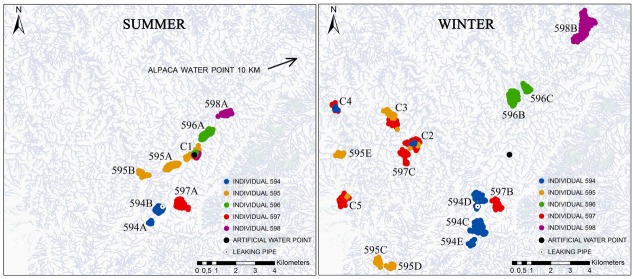
Main recursion sites identified in summer and winter. ‘C’: site common to several of the GPS-monitored individuals.

**Fig 8:**
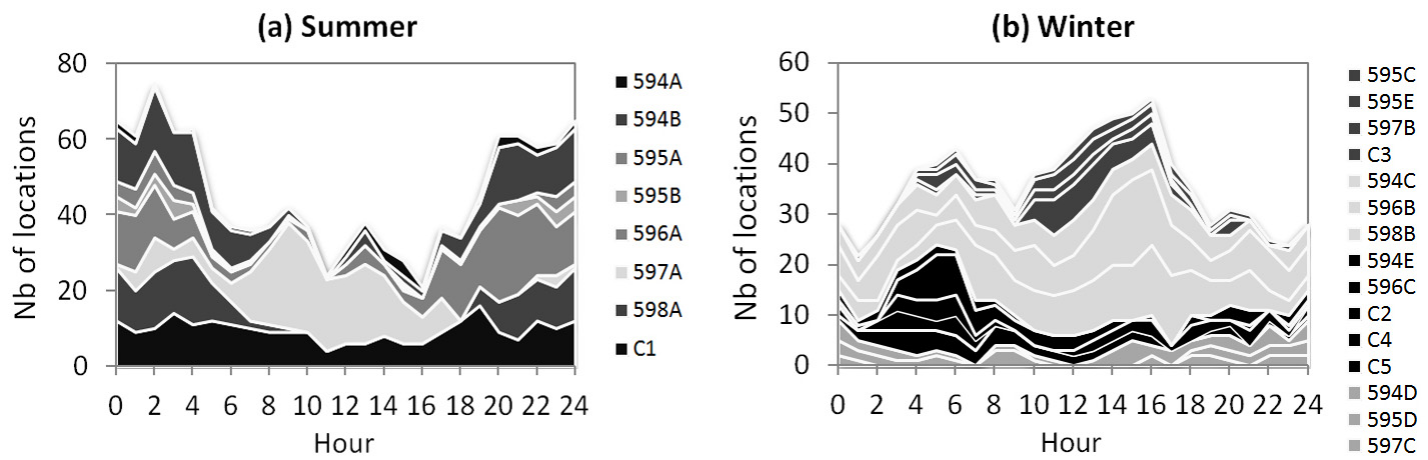
Number of locations in the main recursion sites as a function of the hour of day. Sites are listed in the order in which they appear from top to bottom in the graphs. In winter, the same colour is used for the sites exhibiting similar patterns of frequentation.

In summer, eight main recursion sites were identified (Fig 7), among which one (C1) was common to several individuals. Five of the eight sites (594A, 595B, 596A, 598A, C1) were along or near a streambed, and exhibited high NDVI scores (Table 3); they were used at night (Fig 8a), with the exception of site C1, which encompassed the artificial water point and was visited at all times of the day. The three remaining sites (594B, 595A, 597A) were located on elevated dusty grounds with low NDVI scores: site 594B was south-exposed and included the leaking pipe, site 595A was an almost horizontal plateau, and site 597A was north-exposed. The first two sites (594B, 595A) were visited at night, whereas the latter (597A) was mainly used during the daylight hours (Fig 8a).

**Table 3:** Characteristics of the main recursion sites. For the NDVI, elevation, slope, and northern exposure, values are means (and SD in brackets). In bold: mean (or proportion) higher than the mean (or proportion) in the 95% HR. Sites are listed in the same order as in Fig 8. ‘C’: site common to several of the GPS-monitored individuals.

	Site	Nb locations	Nb visits	NDVI	Thalweg areas	Elevation (m)	Slope (°)	Northern exposure
Summer	594A	33	23	**0.128** (0.013)	**82%**	955 (7)	**7.6** (5.0)	**0.065** (0.054)
	594B	187	86	0.103 (0.004)	13%	**988** (4)	2.6 (2.2)	–0.008 (0.044)
	595A	33	20	0.096 (0.007)	**27%**	**958** (3)	2.0 (1.8)	0.002 (0.029)
	595B	31	18	**0.120** (0.012)	**81%**	**911** (5)	6.0 (4.1)	**0.061** (0.063)
	596A	212	104	**0.136** (0.018)	**83%**	**922** (9)	7.5 (6.3)	**0.073** (0.115)
	597A	222	77	0.104 (0.005)	**49%**	**984** (5)	2.8 (2.5)	**0.030** (0.040)
	598A	140	35	**0.151** (0.029)	**81%**	900 (2)	2.2 (3.0)	**0.022** (0.045)
	C1	227	108	**0.122** (0.010)	**27%**	940 (2)	0.4 (1.3)	0.004 (0.018)
Winter	595C	22	7	0.124 (0.008)	**73%**	849 (12)	**11.3** (4.6)	–0.020 (0.116)
	595E	30	5	0.121 (0.004)	10%	**909** (11)	**13.7** (6.0)	**0.070** (0.193)
	597B	27	14	0.105 (0.006)	**52%**	**986** (7)	4.4 (2.6)	**0.051** (0.045)
	C3	44	21	0.121 (0.009)	**23%**	**897** (7)	4.4 (3.6)	–0.035 (0.068)
	594C	111	17	0.120 (0.011)	**57%**	**960** (19)	8.8 (7.2)	**0.040** (0.165)
	596B	181	72	0.125 (0.014)	**50%**	907 (17)	**9.6** (5.9)	**0.072** (0.116)
	598B	172	40	**0.144** (0.011)	20%	855 (28)	**12.0** (6.0)	**0.059** (0.112)
	594E	25	7	**0.132** (0.004)	**76%**	**907** (13)	**11.2** (4.2)	–0.129 (0.066)
	596C	56	30	**0.132** (0.011)	**52%**	906 (8)	**12.3** (5.2)	–0.029 (0.161)
	C2	39	23	0.120 (0.014)	**54%**	**916** (16)	**14.0** (3.8)	**0.086** (0.145)
	C4	23	15	**0.142** (0.014)	**83%**	767 (5)	5.4 (3.5)	**0.042** (0.071)
	C5	48	9	**0.130** (0.007)	**65%**	**958** (16)	10.7 (5.3)	**0.055** (0.106)
	594D	43	17	0.105 (0.010)	**51%**	**972** (14)	5.0 (2.7)	**0.059** (0.042)
	595D	25	7	0.126 (0.006)	**72%**	839 (11)	10.1 (5.2)	**0.015** (0.126)
	597C	21	9	**0.131** (0.010)	**33%**	**961** (18)	10.1 (6.5)	–0.028 (0.136)

In winter, 15 main recursion sites were identified (Fig 7), among which four (C2, C3, C4, C5) were common to several individuals. Only two of the 15 sites (594D, 597B) overlapped with a summer site (594B and 597A, respectively); they were the winter sites exhibiting the lowest NDVI scores (Table 3), and site 594D was the only winter site including a known water point. Overall, winter sites were less often north-exposed than summer sites (Table 3). Some occupied rather flat areas (elevated ground: 594D, 596C, 597B, C3; bottom of valley side: C4), but most included an entire hillside or large portions of it, in addition to streambeds and/or hill tops (hence resulting in greater values than in summer for mean slope and SD of elevation, slope, and northern exposure). Hourly patterns of frequentation (Fig 8b) were more diverse than in summer, and without a clear relation to the sites’ vegetation cover or topography. Four sites (595C, 595E, 597B, C3) were mainly used during the midday hours; their main common features were their low to moderate NDVI scores and the rather high elevation. The most visited sites (594C, 596B, 598B) were used at all times of the 24-h cycle (Fig 8b); encompassing entire hillsides, they were among the sites exhibiting the greatest SD values for elevation; their NDVI scores were moderate to high, with a maximum for site 598B. Five sites exhibiting a rather high proportion of thalwegs and moderate (C2) to high NDVI scores (594E, 596C, C4, C5) were mainly used during late night and morning, between 3:00 and 8:00. The last three sites (594D, 595C, 597C) were mainly used from 18:00 to 2:00, with no evidence of any other common features.

## Discussion

The findings reported in the present paper must certainly be considered with some caution because of the small number of GPS-monitored animals. Nevertheless, the results obtained are consistent among individuals, and often with what was expected. Accordingly, we are confident that they give quite a reliable picture of the way in which the Asiatic wild ass uses the landscape in the Negev Highlands.

### Home ranges

Because of the low forage availability and renewal in summer, we expected the 50-day home ranges to be greater in summer than in winter (prediction 1). Actually, home ranges tended to be smaller and were closer to the main water point in summer than in winter. In several mobile grazers, the dry-season food shortage tends to induce an increase in home range size [44,71,72]. Summer range contraction around the main water point is consistent with the need to reach a water point on a daily basis ([28,40,48], this study), and confirms that availability of water is an important factor determining space use of the Asiatic wild ass in the Negev Desert. Furthermore, since home range size depends, in addition to environmental factors, on the individual’s characteristics and internal state [73,74], home range contraction is consistent with the tendency of adult males to become territorial in summer [28,29,40,75,76], and thus to restrict one another’s movements.

### Displacement and activity

The distances travelled by the GPS-monitored individuals and the activity level of the observed animals were found to peak at dawn and dusk in both summer and winter. Similar results have been reported for wild ass populations in China [30] and Mongolia [77]. In summer, the twilight peaks co-occurred with switches in habitat use, and the dusk peak further coincided with a maximum in the frequentation of the main water point. In winter, the use of permanent water points was low, and the twilight peaks only corresponded to switches in habitat use. Actually, crepuscular peaks of displacement have been reported in many large mammalian herbivores, including water-independent species [78–80]; and generally speaking, these peaks seem to be elicited by dawn and dusk luminosity or change in luminosity [81]. In this framework, only peak amplitude would be modulated by the need of the animals to join distant water points or habitats on a daily basis.

Because of greater displacements at dawn, dusk and during night in summer, the mean distance travelled was greater in summer than in winter, supporting our second prediction. Furthermore, the locations recorded at night in summer corresponded to favourable feeding areas (high NDVI scores), suggesting the monitored animals covered great distances while feeding in summer. This is consistent with Loarie et al. [82] and Owen-Smith et al. [7], and with observations performed on the Asiatic wild ass in China [30], where individuals move extensively during periods of limited forage condition in order to cover their nutritional needs.

As expected on the basis of ambient temperature (predictions 3 and 4), activity and displacements were reduced during midday in summer. However, the distances covered during the daylight hours differed little between summer and winter, suggesting that the activity level recorded in summer middays was not necessarily induced by temperature but might, as the crepuscular peaks, be mainly linked to ambient luminosity. Nevertheless, as strongly suggested by our results and pointed out by Xia et al. [30], decrease in forage abundance and quality in summer leads the wild ass to spend a considerable amount of time feeding and/or searching for food to reach nutritional requirements at that season. In this context, the low activity level recorded in summer during the daylight hours remains noteworthy.

### Habitat selection

As expected (prediction 5), when midday temperatures were high in summer, the monitored individuals selected high elevations, which are windy, and northern exposures, which receive less sun radiation than other parts of the landscape. The selected areas further exhibited moderate NDVI scores, confirming that the search for cooler microhabitats rather than forage was the main driver of habitat selection during summer middays. Windy areas might also reduce insect harassment [30,83–85]; however, fly abundance and its possible influence on the wild ass’s spatial behaviour were not investigated during the present study, and this option needs further investigation. During winter middays, the monitored animals also selected rather high elevations. However, the selected areas tended to have lower slopes and to be less north-exposed than expected by chance. This preference could be related to the sun exposure and/or to dusty soil presence in elevated grounds, and is consistent with previous study on feral asses (*Equus africanus*; [86]) and with our observations of midday winter groups standing in the sun, rolling, and intensively interacting on high-elevation dusty plateaus and ridges.

In accordance with prediction (6), the monitored animals in summer were mainly found selecting areas with high NDVI scores at night. These areas were located in valleys of moderate elevation which probably exhibited high temperatures preventing their use during the midday hours. No clear hourly pattern emerged in winter in the use of vegetation cover. This is a probable consequence of the more uniform distribution of vegetation over the landscape and the less constraining midday temperatures. Furthermore, vegetation cover was less strongly selected in winter than in summer. Though unanticipated, this result is not really surprising. Vegetation is not only more abundant but also more diverse in winter, and the wild ass probably becomes a more selective grazer in such conditions.

During winter nights, the monitored animals tended to select thalwegs of low to moderate elevation. It might occur for thermal comfort, as the temperature of winter nights is low in the Negev Highlands, and streambeds are wind-sheltered locations [87]. It might also be due to the increased dew availability on vegetation during the night [87–89], in which case it would contribute to lower drinking needs during the winter season [90].

### Recursion movement pattern

The distribution of recursion times revealed peaks of recursion at 24-h intervals, showing that the monitored individuals tended to revisit sites at the same times of the day (prediction 7). A similar pattern has been reported in the red deer (*Cervus elaphus*) by Adrados [91] and in the impala (*Aepyceros melampus*) by Riotte-Lambert et al. [13], and we suspect that 24-h periodicity in recursion movement is a widespread phenomenon among large herbivores.

As expected (prediction 8), recursion movements were performed at different rates according to the season. A higher recursion rate was found in summer despite the low forage availability and renewal. Home range contraction around the main water point certainly contributed to the high recursion rate recorded in summer; as could be expected, the mean number of visits per location was found to be inversely proportional to home range size. However, the difference between seasons was greater than expected only on the basis of home range size. The summer and winter ranges were not used in the same way, as is evidenced by the ratio of the 50% to the 95% home range size (ca. 0.13 in summer against 0.23 in winter) and the number of main recursion sites identified for each season.

We expected that in summer, because of the very low resource renewal, the main recursion sites would be resting sites rather than feeding sites (prediction 9). In contrast to this prediction, most of the main recursion sites identified in summer were favourable feeding sites, exhibiting high NDVI scores, and were located along thalwegs. The assumption deriving from optimal foraging theory and according to which recursions should be a function of time elapsed since last use to allow for resource recovery [15] is probably irrelevant for the dry season in arid environments. Vegetation is especially scarce and heterogeneously distributed in the Negev Desert during the dry season [33], and in this context, the main reason for returning to a previously visited feeding site is likely not regrowth, but the fact that it still provides some forage in a landscape otherwise largely devoid of trophic resources.

In winter, main recursion sites could hardly be classified into foraging or resting sites on the basis of their ecological characteristics. Because of the rather uniform distribution of vegetation, most sites exhibited medium to high NDVI scores. In addition, most of them included valley sides and thus exhibited substantial differences in elevation. Actually, the winter recursion sites including the highest numbers of locations were used at all times of the 24-h cycle, the animals tending to use the top of the sites during the midday hours and their bottom at night, sometimes in the course of a single day. It should be noted, however, that several sites exhibiting high NDVI scores and a high proportion of thalwegs were visited during the second half of the night and at dawn, suggesting they might be primarily used for dew.

In summer, the main recursion sites with low vegetation cover either included access to water and a particular ground texture appreciated for rolling or were characterised by high elevation and visited during the midday hours, implying that their microhabitats were favourable for resting and relieving thermal stress. The frequency of recursion to resting sites was shown to be affected by parasite load in the yellow baboon (*Papio cynocephalus*; [19]). Since parasite density decreases with time in the absence of animals [92], the renewal resource of resting sites is proposed to be the ‘cleanliness’ of an area with regard to parasites. However, the potential effect of parasites on recursion patterns should be further explored.

In winter, the two main recursion sites exhibiting the lowest NDVI scores were part of summer territories, suggesting a fidelity to these territories. Indeed, the two males involved, who defended the core area of their respective home ranges in summer, were observed switching to wintertime multi-male association on larger home ranges, occasionally interrupted by solitary trips to their summer territory (NG, pers. obs.). Finally, only one recursion site in winter included a known water point, suggesting (as do range expansion and the low number of visits to the water points in winter) that water dependency is a less crucial driver of the wild ass’ space use in winter.

### Conclusions and Implications

In the present study, we used a combined approach, integrating movement (displacement and recursions), activity patterns and habitat selection analyses, for exploring the space-use patterns of a water-dependent, desert-dwelling herbivore. This combined approach provided complementary insights: (a) Two main habitat types were found to be selected in both summer and winter: high-elevation areas during the day, and thalweg surroundings during the night. (b) In summer, the former habitat type (almost devoid of vegetation but providing a cooler microclimate) was mostly used for resting, whereas the latter (covered by vegetation) was likely used for feeding. In winter, activity was less clearly related to habitat as a probable consequence of the more uniform vegetation cover and the less constraining midday temperatures. (c) In summer, frequently revisited sites included water sources, but also potential feeding areas despite the very low vegetation renewal. In winter, recursion rates were lower than in summer despite the vegetation dynamics, and recursion sites were more multifunctional (i.e., used for both feeding and resting). In addition, some of the sites frequently revisited in winter resulted from the regular returns of males to their respective summer territories. (d) Finally, in both seasons, the distribution of recursion times revealed a 24-hour periodicity, consistent with the hourly patterns of habitat selection highlighted.

Beyond the case of the Asiatic wild ass, the present study questions the relevance, for the arid environments, of the assumption according to which recursion to a feeding site should be a function of the time required for resource renewal. Furthermore, it points out an intrinsic link between home range size and recursion level; though rather loose, this link implies that we should expect high recursion rates when home range size is restricted because of social factors (such as territorial interactions) or environmental constraints (the rarity of water sources in our study). The 24-hour periodicity that we found in the distribution of recursion times has also been reported in some ruminants [13,91], suggesting it could be a pattern widespread among large mammalian herbivores. This 24-h periodicity is probably related to the tendency of the animals to return to known areas [16,93] and reiterate the same behaviour at the same place [94,95]. It can therefore be expected to generally co-occur with switches in habitat use in the course of the day, but exceptions are possible when habitats are favourable to both feeding and resting. Finally, from a methodological point of view, the present study illustrates the potential advantage of a combined approach integrating movement and habitat selection analyses for inferring the effect of landscape variables and seasonality on space-use patterns. This approach can be applied to studying the space-use patterns of various species living in heterogeneous landscapes. The information gained from such a research could serve as a basis for landscape planning and management regimes, such as nature reserve design, that aim at protecting the studied species.
